# An Optimized Fractional Grey Prediction Model for Carbon Dioxide Emissions Forecasting

**DOI:** 10.3390/ijerph18020587

**Published:** 2021-01-12

**Authors:** Yi-Chung Hu, Peng Jiang, Jung-Fa Tsai, Ching-Ying Yu

**Affiliations:** 1 College of Management & College of Tourism, Fujian Agriculture and Forestry University, Fuzhou 350002, China; ychu@cycu.edu.tw; 2 Department of Business Administration, Chung Yuan Christian University, Taoyuan 32023, Taiwan; 3 School of Business, Shandong University, Weihai 264209, China; 4 Department of Business Management, National Taipei University of Technology, Taipei 10608, Taiwan; jftsai@ntut.edu.tw; 5 College of Management, Yuan Ze University, Taoyuan 32003, Taiwan; mikoyu@saturn.yzu.edu.tw

**Keywords:** genetic algorithm, grey theory, forecasting, fractional-order, carbon dioxide emissions

## Abstract

Because grey prediction does not demand that the collected data have to be in line with any statistical distribution, it is pertinent to set up grey prediction models for real-world problems. GM(1,1) has been a widely used grey prediction model, but relevant parameters, including the control variable and developing coefficient, rely on background values that are not easily determined. Furthermore, one-order accumulation is usually incorporated into grey prediction models, which assigns equal weights to each sample, to recognize regularities embedded in data sequences. Therefore, to optimize grey prediction models, this study employed a genetic algorithm to determine the relevant parameters and assigned appropriate weights to the sample data using fractional-order accumulation. Experimental results on the carbon dioxide emission data reported by the International Energy Agency demonstrated that the proposed grey prediction model was significantly superior to the other considered prediction models.

## 1. Introduction

The International Energy Agency (IEA) [1] reported that carbon dioxide (CO_2_), whose gross emissions in 2019 were 33 billion tons across the globe, is a greenhouse gas that has a direct effect on climate change. There were causes that gave rise to CO_2_ emissions going down in 2019, including the increased consumption of renewable energy in advanced economies such as the European Union, the US, and Japan, and the slowed-down economic growth of emerging markets. Even so, an increase of CO_2_ emissions arising from global economic growth may be still inevitable [1]. Indeed, Asian countries have contributed a huge amount of CO_2_ emissions via fuel combustion. To reduce the negative impact of CO_2_ emissions on the environment and economic growth, it is necessary for national authorities to leverage information derived from forecasting CO_2_ emissions to devise energy-development policies. For instance, China is the highest carbon-emitting country and is facing tremendous pressure to reduce carbon emissions. CO_2_ emissions are expected to go down by 18% in 2020 due to China’s “13th Five-Year Plan” [2].

Many methods, including artificial intelligence techniques such as evolutionary algorithms [3,4], neural networks (NNs) [5,6], and statistical methods such as logistic equations [7], regression models [4,7,8], time series models [9], and the ARIMA model [4,10], have been frequently applied to forecasting. However, the forecasting accuracy of artificial intelligence techniques can be influenced significantly by the training sample size [11], and statistical methods usually require a large amount of data that conform to some statistical assumptions [12]. The grey prediction model was constructed to avoid the inherent flaws arising from the statistical analysis [13]. Grey prediction needs limited data, and it does not demand the collected data to be in line with any statistical properties [13,14]. Grey system theory is an artificial intelligence technique [15], and GM(1,1) has been one of the most commonly used prediction models [13,14,16] among grey systems.

Among the diverse applications of real-world problems analyzed by grey prediction, CO_2_ emissions forecasting is an important issue. GM(1,1) and its variants have been widely applied to forecast CO_2_ emissions, such as the original GM(1,1) by Lin et al. [17], the nonlinear grey Bernoulli model (NGBM(1,1)) by Pao et al. [18], the grey Verhulst model by Wang and Li [19], and the adaptive grey model by Xu et al. [20]. In addition to GM(1,1), the multivariate model GM(1,N), comprised of N-1 relevant factor sequences and a characteristic sequence, is often applied to CO_2_ emission forecasting, such as the nonlinear multivariable models of Wang and Ye [21] and Wu et al. [22] and the grey multivariable model based on the trends of driving variables of Ding et al. [23]. Compared with GM(1,1), the motivation of using GM(1,N) arises from the fact that multivariate techniques may improve forecasting ability [24,25]. Despite the usefulness of grey prediction, several issues arising from the above-mentioned grey prediction models motivated us to further develop an effective grey prediction model for forecasting CO_2_ emissions.

First, the performance of multiple variables models, such as econometric methods, can be adversely affected if we know little about the relevant explanatory factors [26]. Despite CO_2_ emissions being mainly influenced by economic development, population, and energy use [23], we do not know all of the relevant factors. This led us to consider GM(1,1) as a development base in this study, rather than GM(1,*N*). Next, when using GM(1,1) or one of its variants, potential regularities embedded in data sequences are usually recognized by the one-order accumulated generating operation (1-AGO). A problem arising from 1-AGO is that each sample is treated with equal weighting [27]. Thus, it is pertinent to consider FAGM(1,1) by incorporating fractional-order accumulation into GM(1,1) to mitigate such a restriction [23,24]. Several variants have been proposed to strengthen the FAGM(1,1), such as fractional NGBM(1,1) (FANGBM(1,1)) by Wu et al. [22] and Şahin [28] and fractional GM(*q*,1) by Mao et al. [29]. Despite the usefulness of fractional-order accumulation, limited studies related to grey prediction, apart from the fractional time-delayed grey model (FTDGM) of Ma et al. [30], the nonhomogeneous grey model of Wu et al. [31], and the discrete fractional GM(1,1) by Gao et al. [32], have addressed CO_2_ emission forecasting using FAGM(1,1).

The last issue we address here is that FAGM(1,1) and its variants usually require application of the ordinary least square (OLS) method to derive the control variable and developing coefficient by means of background values, which are not easily determined [33,34]. Thus, this study proposes a genetic algorithm (GA)-based fractional grey prediction model (GA-FAGM(1,1)) to determine relevant parameters without background values. The usefulness and applicability of GA-FAGM(1,1) are verified via its application to annual CO_2_ emission forecasting. Compared with the other considered prediction models, the results demonstrate that GA-FAGM(1,1) performs well.

The remainder of this paper is organized as follows. Section 2 introduces the original GM(1,1) and its fractional version. The proposed GA-FAGM(1,1) is described in Section 3. Section 4 examines the CO_2_ emissions forecasting accuracy of the different considered prediction models. A discussion and conclusions are presented in Section 5.

## 2. GM(1,1) and Fractional GM(1,1)

### 2.1. GM(1,1) Model

Consider an original sequence $x_{}^{\left(0\right)}$ = ($x_{1}^{\left(0\right)}$,$x_{2}^{\left(0\right)}$,…,$x_{n}^{\left(0\right)}$) composed of *n* data samples. $x_{}^{\left(1\right)}$ = ($x_{1}^{\left(1\right)}$,$x_{2}^{\left(1\right)}$,…,$x_{n}^{\left(1\right)}$), which is further derived by 1-AGO as

(1) $$x_{k}^{\left(1\right)}\;=\;\sum_{j=1}^{k}x_{j}^{\left(0\right)},\;k\;=\;1,\;2,\text{…},\;n$$

Since $x_{}^{\left(1\right)}$ is monotonically increasing, the whitening equation, which is treated as a mathematical form of the GM(1,1), is expressed as

(2) $$\frac{dx^{\left(1\right)}(t)}{dt}\;+\;{\mathrm{ax}}^{\left(1\right)}(t)=b$$

where *a* is the developing coefficient and *b* is the control variable. The corresponding time response function is obtained by solving the whitening equation as

(3) $${\hat{x}}_{k}^{\left(1\right)}\;=\;(x_{1}^{\left(0\right)}\;-\;\frac{b}{a})e^{-a(k-1)}+\frac{b}{a}$$

It turns out that a linear regression model can be used to estimate *a* and *b* as

(4) $$x_{k}^{\left(0\right)}\;+\;az_{k}^{\left(1\right)}=b,\;k\;=\;2,\;3,\text{…},\;n$$

where *z*^(1)^ = ($z_{2}^{\left(1\right)}$,$z_{3}^{\left(1\right)}$,…,$z_{n}^{\left(1\right)}$) is a sequence of background values, and $z_{k}^{\left(1\right)}$ (*k* = 2, 3,…, *n*) is given by

(5) $$z_{k}^{\left(1\right)}\;=\;\alpha x_{k}^{\left(1\right)}\;+\;(1\;-\;\alpha )x_{k-1}^{\left(1\right)}$$

where α is set to 0.5 commonly. OLS can then be applied to obtain the parameters *a* and *b*:

[*a*, *b*]^*T*^ = (**B**^*T*^**B**)^−1^**B**^*T*^**y** (6)

where

(7) $$B\;=\;\left[\begin{array}{cc} -z_{2}^{\left(1\right)} & 1\\ -z_{3}^{\left(1\right)} & 1\\ \vdots & \vdots \\ -z_{n}^{\left(1\right)} & 1 \end{array}\right],\;y\;=\;\left[\begin{array}{c} x_{2}^{\left(0\right)}\\ x_{3}^{\left(0\right)}\\ \vdots \\ x_{n}^{\left(0\right)} \end{array}\right]$$

which accounts for why *z*^(1)^ has a strong impact on the determination of *a* and *b*. At last, the one-order inverse AGO (1-IAGO) is applied to compute the predicted value of $x_{k}^{\left(0\right)}$ as

(8) $${\hat{x}}_{k}^{\left(0\right)}=\;{\hat{x}}_{k}^{\left(1\right)}\;-\;{\hat{x}}_{k-1}^{\left(1\right)},\;k\;=\;2,\;3,\text{…},\;n$$

However, the above linear regression model might not follow the Gauss–Markov theorem [35]; thereby, the resultant estimators obtained by OLS may not be the best unbiased estimators, which suggests that ${\hat{x}}_{1}^{\left(0\right)}(k)$ may be unreliable.

### 2.2. Fractional GM(1,1) Model

GM*^p^*(1,1) is a form of GM(1,1) that has been combined with a fractional-order accumulator, where *p* is the fractional parameter (0 < *p* < 1). An accumulated generating sequence, $x_{}^{\left(p\right)}$ = ($x_{1}^{\left(p\right)}$,$x_{2}^{\left(p\right)}$,…,$x_{n}^{\left(p\right)}$), with *p*-order is generated by *p*-AGO as:

(9) $$x_{k}^{\left(p\right)}=\sum_{i=1}^{k}\left(\begin{array}{c} k-i+p-1\\ k-i \end{array}\right)x^{\left(0\right)}(i),\;k\;=\;1,\;2,\text{…},\;n$$

where

(10) $$\left(\begin{array}{c} k-i+p-1\\ k-i \end{array}\right)=\frac{(k-i+p-1)(k-i+p-2)\ldots (p+1)p}{(k-i)!}$$

For instance, provided that *n* = 4 and *p* = 0.8, the coefficient of $x_{1}^{\left(0\right)}$ in the generation of $x_{4}^{\left(0.8\right)}$ is computed as

$$\left(\begin{array}{c} 4-1+0.8-1\\ 4-1 \end{array}\right)=\left(\begin{array}{c} 2.8\\ 3 \end{array}\right)=\frac{2.8\times 1.8\times 0.8}{6}$$

It has been proven that *p*-AGO satisfies the so-called principle of new information priority when 0 < *p* < 1. The smaller the value of *p*, the smaller weights older data are assigned [22,27,36]. That is, newer data are more weighted when a smaller value of *p* is given.

The solution to $dx^{\left(p\right)}$(*t*)/*dt* + *a*^(*p*)^*x*^(*p*)^(*t*) *= b*^(*p*)^, i.e., a whitening equation with respect to GM*^p^*(1,1), is given by

(11) $${\hat{x}}_{k}^{\left(p\right)}\;=\;(x_{1}^{\left(0\right)}\;-\;\frac{b^{\left(p\right)}}{a^{\left(p\right)}})e^{-a(k-1)}+\frac{b^{\left(p\right)}}{a^{\left(p\right)}}$$

The original form is expressed as:

(12) $$x_{k}^{\left(p\right)}\;-\;x_{k-1}^{\left(p\right)}\;+\;az_{k}^{\left(p\right)}\;=\;b$$

where *z*^(*p*)^ = ($z_{2}^{\left(p\right)}$,$z_{3}^{\left(p\right)}$,…,$z_{n}^{\left(p\right)}$) is a sequence of background values, and $z_{k}^{\left(p\right)}$ (*k* = 2, 3,…, *n*) is given as:

(13) $$z_{k}^{\left(p\right)}\;=\;\alpha x_{k}^{\left(p\right)}\;+\;(1\;-\;\alpha )x_{k-1}^{\left(p\right)}$$

where *α* is usually set to 0.5. OLS can be applied to derive *a*^(*p*)^ and *b*^(*p*)^ in the case where *α* and *p* are given

(14) $$B\;=\;\left[\begin{array}{cc} -z_{2}^{\left(p\right)} & 1\\ -z_{3}^{\left(p\right)} & 1\\ \vdots & \vdots \\ -z_{n}^{\left(p\right)} & 1 \end{array}\right],\;y\;=\;\left[\begin{array}{c} x_{2}^{\left(p\right)}-x_{1}^{\left(p\right)}\\ x_{3}^{\left(p\right)}-x_{2}^{\left(p\right)}\\ \vdots \\ x_{n}^{\left(p\right)}-x_{n-1}^{\left(p\right)} \end{array}\right]$$

Furthermore, it is clear that *z*^(*p*)^ determines *a*^(*p*)^ and *b*^(*p*)^. In common with GM(1,1), we cannot guarantee that the linear regression model follows the Gauss–Markov theorem. To obtain ${\hat{x}}_{k}^{\left(0\right)}$, we first apply (1 − *p*)-AGO to (${\hat{x}}_{1}^{\left(p\right)}$,${\hat{x}}_{2}^{\left(p\right)}$,…,${\hat{x}}_{n}^{\left(p\right)}$) to obtain an accumulated generating sequence, (${\hat{x}}_{1}^{\left(1\right)}$,${\hat{x}}_{2}^{\left(1\right)}$,…,${\hat{x}}_{n}^{\left(1\right)}$), with (1 − *p*)-order. Next, 1-IAGO is applied to (${\hat{x}}_{1}^{\left(1\right)}$,${\hat{x}}_{2}^{\left(1\right)}$,…,${\hat{x}}_{n}^{\left(1\right)}$) to obtain (${\hat{x}}_{1}^{\left(0\right)}$,${\hat{x}}_{2}^{\left(0\right)}$,${\hat{x}}_{n}^{\left(0\right)}$).

## 3. The Proposed Optimized Grey Prediction Model

**Figure 1:**
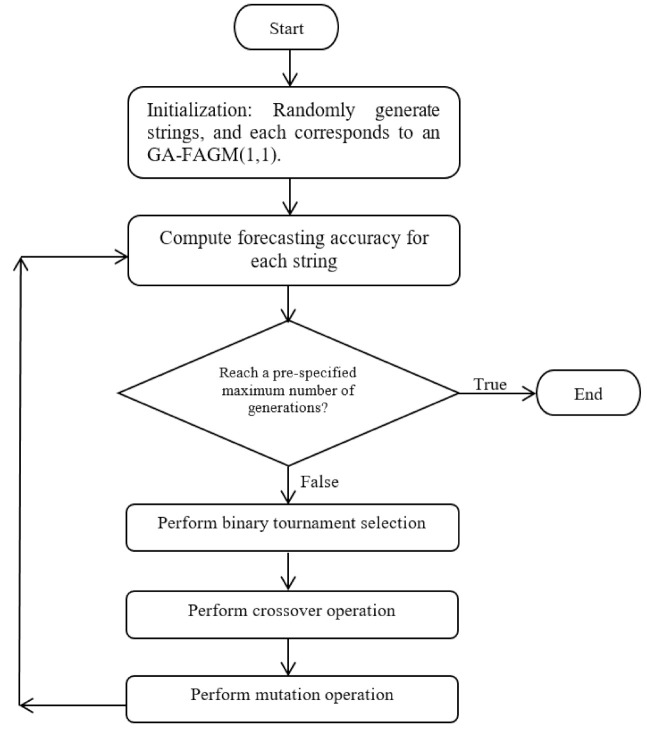
Flowchart of a genetic algorithm (GA) for constructing GA-FAGM(1,1).

Although ${\hat{x}}_{k}^{\left(p\right)}$ in the original FAGM(1,1) can be obtained by the relevant parameters, including the fractional parameter, *p*, the developing coefficient, *a*^(*p*)^, and the control variable, *b*^(*p*)^, it is not required to apply OLS to derive *a*^(*p*)^ and *b*^(*p*)^ to avoid the problems arising from the Gauss–Markov theorem. This leads us to find relevant parameters of FAGM(1,1) using a GA instead of OLS. A flowchart of constructing the proposed GA-FAGM(1,1) is depicted in Figure 1.

### 3.1. Problem Formulation

To develop an optimized prediction model, the mean absolute percentage error (MAPE) is used to formulate the objective of our problem as

(15) $$\mathrm{Minimize}\;\mathrm{MAPE}\;=\;\frac{1}{n}\sum_{k=1}^{n}\frac{\left|x_{k}^{}-{\hat{x}}_{k}^{}\right|}{x_{k}^{}}\times 100\%$$

where *x_k_* and ${\hat{x}}_{k}^{}$ are the actual and forecasted values at time *k* (*k* = 1, 2,…, *n*), respectively. MAPE has become a benchmark to evaluate prediction accuracy since it has been proven that MAPE is more stable than other commonly used measures, including the root mean square error (RMSE) and mean absolute error (MAE) [37]. Dang et al. [38] demonstrated the effectiveness of MAPE when constructing optimized grey prediction models as well. We thus use MAPE to evaluate the fitness of a chromosome.

### 3.2. Coding

Three required parameters (i.e., *a*^(*p*)^, *b*^(*p*)^, and *p*) can be discovered by a GA. A chromosome consisting of *a*^(*p*)^, *b*^(*p*)^, and *p* in a population corresponds to GA-FAGM(1,1) such that smaller fitness values produce better chromosomes. To align with the new information priority principle, *p* ranging from zero to 1 is considered because *p*-AGO can discriminate in favor of older data as *p* > 1.

### 3.3. Genetic Operations

Let *n_max_* and *n_size_* denote the maximum number of generations and the population size, respectively. Selection, crossover, and mutation are applied to generate *n_size_* new chromosomes for *P_m_*_+1_ after the fitness value of each string has been evaluated for *P_m_*, where *P_m_* denotes a population in the *m*-th generation (1 ≤ *m* ≤ *n_max_*). When *P_m_* is treated as the current population, *P_m_*_+1_ is the next population for *P_m_*.

#### 3.3.1. Selection

Two strings are selected randomly from *P_m_* by binary tournament selection with replacement to generate new strings in *P_m_*_+1_. The string with higher fitness is thereby put in the mating pool. We end the whole learning process when *n_size_* strings have been placed in the mating pool.

#### 3.3.2. Crossover and Mutation

From the mating pool, the parent strings, say *u* ($p_{u}^{m}$ $a_{u}^{\left(p\right)}$ $b_{u}^{\left(p\right)}$) and *v* ($p_{v}^{m}$ $a_{v}^{\left(p\right)}$ $b_{v}^{\left(p\right)}$) (1 ≤ *u*, *v* ≤ *n_size_*), are selected, and crossover and mutation are applied to reproduce children. The crossover ends up with a generation of offspring including *u*′ (${p_{u}^{m}}^{\prime }$ ${a_{u}^{\left(p\right)}}^{\prime }$ ${b_{u}^{\left(p\right)}}^{\prime }$) and *v*′ (${p_{v}^{m}}^{\prime }$ ${a_{v}^{\left(p\right)}}^{\prime }$ ${b_{v}^{\left(p\right)}}^{\prime }$) by employing each pair of parameters in *u* and *v* with crossover rate *Pr*_c_ as:

(16) $${p_{u}^{m}}^{\prime }\;=\;\alpha_{1}p_{u}^{m}\;+\;(1\;-\alpha_{1})p_{v}^{m},\;{p_{v}^{m}}^{\prime }\;=\;(1\;-\;\alpha_{1})\;p_{u}^{m}\;+\;\alpha_{1}p_{v}^{m}$$

(17) $${a_{u}^{\left(p\right)}}^{\prime }\;=\;\alpha_{2}a_{u}^{\left(p\right)}\;+\;(1\;-\;\alpha_{2})a_{v}^{\left(p\right)},\;{a_{v}^{\left(p\right)}}^{\prime }\;=\;(1\;-\;\alpha_{2})a_{u}^{\left(p\right)}\;+\;\alpha_{2}a_{v}^{\left(p\right)}$$

(18) $${b_{u}^{\left(p\right)}}^{\prime }\;=\;\alpha_{3}b_{u}^{\left(p\right)}\;+\;(1\;-\;\alpha_{3})b_{v}^{\left(p\right)},\;b_{v}^{\left(p\right)}\prime \;=\;(1\;-\;\alpha_{3})b_{u}^{\left(p\right)}\;+\;\alpha_{3}b_{v}^{\left(p\right)}$$

where *α*_1_, *α*_2_, and *α*_3_ are random numbers in the unit interval.

In a newly generated string, a tiny positive or minus value, is added to alter a parameter with mutation rate *Pr_m_*. A higher value of *Pr_c_* is often recommended because it benefits the exploration of more solution space. Furthermore, *Pr_m_* should be set to a lower value to prevent the evolution from excessive perturbations [39].

#### 3.3.3. Elitist Strategy

For *P_m_*_+1_, the elitist strategy intends to retain strings with high fitness from *P_m_*. Indeed, only a few elite strings are enough to generate good results [39]. In *P_m_*, a string with a minimum fitness serves as an elite string. *n_del_* (0 ≤ *n_del_* ≤ *n_size_*) strings can be eliminated from *P_m_*_+1_ randomly, and the elite strings are then added to *P_m_*_+1_ *n_size_* times.

### 3.4. Algorithm Design

The proposed GA-FAGM(1,1) is set up by employing the GA to optimize the relevant parameters. The pseudocode corresponding to the GA for constructing the GA-FAGM(1,1) is described as follows:
*m*←1;
Generate *n_size_* strings in *P_m_*; //Initialization//
while *m* < *n_max_* do
   Compute the fitness value of each string in *P_m_*;
   Choose the strings with top *n_del_*fitness to be elites;
   repeat
     Randomly choose two strings from *P_m_*;
     Put the string with higher fitness in the mating pool;
   until *n_size_* strings in the mating pool;
   repeat
     Select the parent strings *u* and *v* from the mating pool;
     Perform crossover to generate new parameters; //Equations (17)–(19)//
     Add a tiny positive/negative value to each new parameter; //mutation//
     Add offspring *u*′ and *v*′ to *P_m_*_+1_;
   until *n_size_* strings in *P_m_*_+1_;
   Randomly remove *n_del_* strings from *P_m_*_+1_;
   Add *n_del_* elites to *P_m_*_+1_; //elitist strategy//
   *m*←*m* + 1;
end.

## 4. Applications of CO_2_ Emissions Forecasting

Reduction of the negative impact of emissions of CO_2_ on the global environment and economic growth is urgently needed. One way to achieve this goal is to develop prediction models with high accuracy for forecasting CO_2_ emissions; indeed, such an approach has become increasingly important for national authorities.

### 4.1. Comparative Prediction Models

Here, the GA-FAGM(1,1) is compared to the other considered grey prediction models as follows:

(1) GM(1,1): To find the optimal *α*, the Linear Interactive and General Optimizer is applied to set up an optimized GM(1,1) with OLS by minimizing MAPE [38].

(2) FAGM(1,1): OLS can be used to set up FAGM(1,1) by minimizing MAPE to find the optimal *α* and *p* (0 ≤ *α* ≤ 1, 0 < *p* < 1). Note that, compared to the original versions of GM(1,1) and FAGM(1,1), the control variable and the developing coefficient can be directly determined by GA-FAGM(1,1) without using background values.

(3) FANGBM(1,1) [2]: A *p*-order differential equation serves as the mathematical form of FANGBM(1,1), which is given by:

(19) $$\frac{dx^{\left(p\right)}(t)}{dt}\;+\;a^{\left(p\right)}x^{\left(p\right)}(t)=b{\left(x^{\left(p\right)}(t)\right)}^{r},\;r\neq 1$$

The time response function is

(20) $${\hat{x}}_{k}^{\left(p\right)}\;=\;[((x_{1}^{\left(0\right)}{)}^{1-r}-\frac{b^{\left(p\right)}}{a^{\left(p\right)}})\;e^{-a^{\left(p\right)}(k-1)(1-r)}+\frac{b}{a}{]}^{1/(1-r)}$$

As *α*, *p*, and *r* are given, OLS is applied to derive *a*^(*p*)^ and *b*^(*p*)^. The optimal values of *α*, *p*, and *r* are thereby determined by a GA with the minimization of MAPE.

(4) FTDGM [30]: The whitening equation of FTDGM is given by:

(21) $$\frac{dx^{\left(p\right)}(t)}{dt}\;+\;a^{\left(p\right)}\;x^{\left(p\right)}(t)=b^{\left(p\right)}\;t^{\left(p\right)}+c,\;r\neq 1$$

The time response function is

(22) $${\hat{x}}_{k}^{\left(p\right)}\;=\;(x_{1}^{\left(0\right)})e^{-a^{\left(p\right)}(k-1)}\;+\;\sum_{i=2}^{k}e^{-a^{\left(p\right)}(k-i+\frac{1}{2})}\frac{\left(f(i)+f(i-1)\right)}{2}$$

where

*f*(*i*) = *bi*^(*p*)^ + *c* (23)

(24) $$i^{\left(p\right)}\;=\;\sum_{j=1}^{i}\left(\begin{array}{c} i-j+p-1\\ k-j \end{array}\right)j$$

As *α* and *p* are given, OLS is applied to derive *a*^(*p*)^ and *b*^(*p*)^. Using *α* = 0.5, a GA is employed to determine the optimal value of *p* that can minimize MAPE.

To construct the optimized versions of FAGM(1,1), FANGBM(1,1), FTDGM, and GA-FAGM(1,1), GAs are implemented for individual grey prediction models to find the relevant parameters optimally, in which *n_max_*, *n_size_*, *p_c_*, and *p_m_* are set to 1000, 200, 0.9, and 0.01, respectively. For instance, *α*, *p*, and *r* relevant to FANGBM(1,1) can be automatically determined by a GA. Commonly used forecasting models, including NNs, fuzzy time series analysis (FTS), and the ARIMA model, were considered as well. The related parameter specifications for training a NN included one hidden layer with five hidden nodes, a learning rate of 0.5, and ten thousand repetitions. The average prediction accuracy of a NN was computed after performing ten independent trials.

As for the FTS analysis, the computational steps are briefly introduced as follows [40]:

(1) On the basis of the minimum and maximum values of the available data, a universe of discourse *U* is defined. Then, *U* is equally divided up into *s* subintervals using *s*+1 partitioning points (*p*_1_, *p*_2_,…, *p_s_*_+1_).

(2) Create *s*+1 triangular fuzzy sets, *A*_1_, *A*_2_,…, *A_s_*_+1_ such that *A*_1_ = (*p*_1_, *p*_1_, *p*_2_), *A*_2_ = (*p*_1_, *p*_2_, *p*_3_),…, *A_s_*_+1_ = (*p_s_*, *p_s_*_+1_, *p_s_*_+1_).

(3) To generate an FTS denoted by *F*(*t*), $x_{j}^{\left(0\right)}$ (1 ≤ *j* ≤ *n*) is fuzzified to *A_l_*_+1_ when $\mu_{A_{l}}(x_{j}^{\left(0\right)})$ ≤ $\mu_{A_{l+1}}(x_{j}^{\left(0\right)})$; otherwise to *A_l_* (1 ≤ *l* ≤ *s*).

(4) Let *F*(*t* − 1)→*F*(*t*) denote a fuzzy logical relationship *F*(*t*−1)→*F*(*t*), which means that *F*(*t*) is caused by *F*(*t* − 1). In the FTS, we employ *A_r_* →*A_q_* (*v*) to represent the case where *A_r_* →*A_q_* (1 ≤ *q*, *r* ≤ *s* + 1) appears *v* times.

(5) In the case of *F*(*t* − 1) = *A_r_*, and at least two fuzzy logical relationships, $A_{r}\to A_{q_{1}}\;(v_{q_{1}})$, $A_{r}\to A_{q_{2}}\;(v_{q_{2}})$,…, $A_{r}\to A_{q_{z}}\;(v_{q_{z}})$ (1 ≤ *q*_1_, *q*_2_,…, *q_z_* ≤ *s* + 1) are available at time *t* − 1. The predicted value is 0.5(${\bar{x}}_{r}$ + ${\bar{x}}^{\prime }$), where ${\bar{x}}_{r}$ and ${\bar{x}}^{\prime }$ can be expressed, as appears *v* times

(25) $${\bar{x}}_{r}=\{\begin{array}{l} \frac{p_{1}\;+\;0.5m_{1}}{1.5},\;\mathrm{if}\;r=1\\ \frac{0.5m_{r-1}\;+\;p_{r}\;+\;0.5m_{r+1}}{2},\;\mathrm{if}\;r=2,\ldots ,s\\ \frac{0.5m_{s}\;+\;p_{s+1}}{1.5},\mathrm{if}\;r=s+1 \end{array}$$

(26) $${\bar{x}}^{\prime }=\frac{v_{1}\times {\bar{x}}_{q_{1}}+v_{2}\times {\bar{x}}_{q_{2}}+\ldots +v_{z}\times {\bar{x}}_{q_{z}}}{v_{1}+v_{2}+\ldots +v_{z}}$$

where *m_r_* is the midpoint of interval (*p_r_*, *p_r_*_+1_). The predicted value is 0.5 (${\bar{x}}_{r}$ + ${\bar{x}}_{q}$), as only *A_r_* →*A_q_* is available. However, the predicted value is ${\bar{x}}_{r}$ when all FLR at hand are not available for forecasting.

### 4.2. Experimental Results

To examine the forecasting accuracy of the different prediction models considered here, according to statistics from the IEA [1], countries whose total amount of CO_2_ emissions ranked among the top 20 since 2000 were taken into account.

The historical data shown in Table 1 span from 2003 to 2017. This study employs data from 2003 to 2013 for the model fitting and employs the other data for ex-post testing. Table 2 summarizes the results of the ex-post testing. Compared with the other considered prediction models, GA-FAGM(1,1) performed well. Moreover, the proposed GA-FAGM(1,1) performed best for 11 out of 20 data sequences.

To examine the differences among the considered prediction models, the Friedman test with a post-hoc test, namely the Nemenyi test, was employed to statistically analyze the eight prediction models applied to the 20 datasets. Let *r_j_* denote the average rank of prediction model *j* (*j* = 1, 2,…, 8). As seen in Table 2, *r*_1_ = 5.3, *r*_2_ = 5.75, *r*_3_ = 5.1, *r*_4_ = 6.2, *r*_5_ = 4.75, *r*_6_ = 4.05, *r*_7_ = 3.15, and *r*_8_ = 1.7 were obtained for GM(1,1), FAGM(1,1), FANGBM(1,1), FTDGM, NN, ARIMA, FTS, and GA-FAGM(1,1), respectively. The smaller the average rank, the better the forecasting model performed.

Let *k*_1_ be the number of prediction models considered and *k*_2_ be the number of data sequences used. The null hypothesis claims that the ranks of the considered prediction models are, on average, identical. As a result, the null hypothesis is rejected since the Friedman statistic is 10.98 that exceeds the critical value of *F*(*k*_1_ − 1, (*k*_1_ − 1)(*k*_2_ − 1)) (2.08) at the 5% level.

The Nemenyi test was further employed to detect differences by the critical difference (*CD*) expressed as

(27) $$CD=\;3.03\sqrt{\frac{k_{1}(k_{1}+1)}{6k_{2}}}$$

where *CD* equals 2.35 at the 5% level. This means that a prediction model is significantly superior to another model in the case that the average rank of the latter is less than that of the former by *CD*. The results are summarized below:

(1) The proposed GA-FAGM(1,1) had the minimum rank on average and significantly outperformed the other prediction models except for FTS.

(2) Despite the fact that GA-FAGM(1,1) did not significantly outperform FTS, the rank of the former was smaller than the latter. The results showed that the latter performed worse than the former for 15 out of 20 data sequences.

(3) Besides GA-FAGM(1,1), the optimized GM(1,1) was not significantly inferior to the other considered prediction models. Note that GM(1,1) is often treated as a benchmark when comparisons were made among different grey prediction models.

(4) Although FAGM(1,1) was not significantly superior to GM(1,1), it was interesting to note that the average rank of the former was smaller than that of the latter.

## 5. Discussion

As for the computational complexity, we analyzed the time complexities among the different grey prediction models considered in this work. Because the time complexities of OLS, fractional order accumulation, and GA are O(*n*^2^), O(*n*), and O(*n_max_⋅n_size_*), respectively, the time complexities of FAGM(1,1), FANGBM(1,1), and FTDGM are all O(*n*^2^‧*n_max_*⋅*n_size_*), but that of GA-FAGM(1,1) without performing OLS, is O(*n*‧*n_max_*⋅*n_size_*). Therefore, GA-FAGM(1,1) is more efficient than FAGM(1,1), FANGBM(1,1), and FTDGM.

The results found here indicated that the proposed GA-FAGM(1,1) is applicable to other prediction problems as well, such as energy and tourism demand forecasting. Indeed, when endeavoring to set up development plans for energy demand and consumption, the prediction of energy demand has become increasingly noteworthy for government administrations [41], especially in developing countries [42]. Moreover, it has been shown that the residual GM(1,1) improved the forecasting accuracy of GM(1,1) [13,14]. In a similar way, it is possible to develop the residual FAGM(1,1) to improve FAGM(1,1). It turns out that how to construct the residual GA-FAGM(1,1) to improve GA-FAGM(1,1) can be an interesting issue. Additionally, despite incomplete information with respect to relevant factors for the prediction problems with which we are concerned, it could be worth constructing a multivariate grey prediction model by extending GA-FAGM(1,1) to GA-FAGM(1,*N*). These remain the focus of future work.

## 6. Conclusions

In light of the effectiveness and applicability of grey prediction for forecasting CO_2_ emissions, the development of such models appears to be profitable. In the case of leveraging prediction models for CO_2_ emissions, it is helpful for authorities to set up competitive strategies for economic growth and environmental protection by inhibiting CO_2_ emissions.

This study highlighted the usefulness of GA-FAGM(1,1) by incorporating three significant characteristics into the proposed grey prediction model, including the use of single variable model as a development base, fractional order accumulation, and determination of the control variable and developing coefficient without background values. These features make the GA-FAGM(1,1) novel compared to the other fractional grey prediction models considered here.

From Table 2, it can be found that the forecasting accuracy of the proposed GA-FAGM(1,1) for CO_2_ emission forecasting was quite encouraging. With the GA, the proposed GA-FAGM(1,1) significantly outperformed the other grey prediction models considered here. We thereby conclude that the mechanism of determining the relevant parameters makes the proposed prediction model perform significantly better. The experimental results also emphasize the applicability and usefulness of GA-FAGM(1,1) in terms of forecasting CO_2_ emissions.

## Figures and Tables

**Table 1:** Historical annual carbon dioxide emission from top twenty countries (unit: million tons).

Country	2003	2004	2005	2006	2007	2008	2009	2010	2011	2012	2013	2014	2015	2016	2017
China	4068.1	4741.8	5407.5	5961.8	6473.2	6669.1	7131.5	7832.7	8570.9	8819.6	9190.5	9127.2	9101.4	9064.4	9257.9
USA	5610.7	5688.8	5703.2	5602.4	5686.7	5512.5	5120.7	5352.1	5128.2	4903.0	5038.5	5046.6	4928.6	4838.5	4761.3
Russia	1493.9	1488.2	1481.9	1537.7	1533.7	1553.8	1440.7	1529.2	1604.7	1607.9	1568.5	1551.6	1534.5	1510.6	1536.9
India	950.3	1022.3	1073.7	1147.7	1260.9	1338.9	1502.3	1583.4	1667.8	1803.8	1854.8	2018.2	2026.7	2057.7	2161.6
Japan	1174.4	1166.1	1166.8	1144.7	1186.4	1122.3	1070.3	1127.2	1183.5	1225.9	1234.0	1194.1	1155.7	1146.9	1132.4
Germany	820.7	804.7	786.7	799.1	766.7	775.2	720.2	758.8	731.2	744.7	763.8	723.2	729.7	734.5	718.8
Canada	534.3	526.2	540.4	531.1	561.9	541.9	514.4	528.6	541.2	539.7	549.6	555.5	557.7	548.1	547.8
Korea	437.8	459.8	457.7	464.7	477.4	488.8	502.1	550.9	573.8	575.5	574.6	562.7	582.0	589.2	600.0
UK	533.5	533.4	531.6	532.9	521.7	507.9	460.1	476.6	439.2	461.4	447.0	408.7	394.1	372.6	358.7
Iran	357.4	385.9	417.8	449.3	480.1	487.3	504.3	498.6	507.8	512.3	536.0	556.7	553.3	554.4	567.1
Mexico	386.6	396.0	412.4	426.9	433.4	434.5	425.2	440.5	456.5	459.5	449.6	434.2	442.4	446.2	446.0
Italy	445.3	455.0	456.4	449.2	441.5	428.9	383.7	392.0	384.1	366.7	337.6	319.2	329.7	325.7	321.5
South Africa	348.3	375.3	372.3	374.2	391.5	421.6	398.6	418.8	403.4	421.0	430.8	442.5	418.3	418.7	421.7
Saudi Arabia	266.3	282.1	298.0	316.6	333.2	364.3	379.5	419.2	434.6	463.4	471.1	506.7	531.6	526.9	532.2
Australia	348.0	361.3	365.5	370.8	381.1	384.3	391.1	383.6	382.1	381.7	375.8	367.0	373.8	381.9	384.6
Indonesia	308.8	316.0	317.8	339.3	355.4	349.1	360.6	357.6	390.3	415.2	418.0	456.9	459.1	454.3	496.4
Brazil	293.3	310.8	311.6	315.2	330.8	349.3	325.5	372.0	391.1	424.1	453.5	477.8	453.6	418.5	427.6
France	368.3	369.1	371.9	362.6	353.8	349.5	336.1	340.2	322.3	325.3	325.3	293.2	299.6	301.7	306.1
Poland	293.1	296.7	296.3	308.1	306.3	301.6	291.5	307.5	303.2	296.9	292.4	279.3	282.7	293.2	305.8
Spain	302.6	319.3	333.7	325.0	337.9	309.8	276.1	262.1	264.9	260.5	235.2	232.1	247.1	237.4	253.4

**Table 2:** MAPE of different prediction models.

Country	GM(1,1)	FAGM(1,1)	FANGBM(1,1)	FTDGM	NN	ARIMA	FTS	GA-FMGM(1,1)
China	23.15	15.77	12.87	17.11	18.27	3.98	2.27	12.56
USA	2.65	0.87	2.12	4.56	7.85	1.92	1.37	0.90
Russia	5.42	2.70	3.42	7.98	7.56	0.76	1.01	0.71
India	10.18	3.04	1.46	6.31	2.70	3.41	4.48	3.00
Japan	4.52	22.63	15.17	13.43	13.03	0.90	2.82	2.31
Germany	1.15	1.10	1.47	1.81	3.53	3.15	2.04	0.98
Canada	1.72	3.17	2.14	3.58	1.55	2.57	0.78	1.06
Korea	6.94	5.41	14.86	1.47	9.76	1.52	1.29	2.44
UK	9.16	10.59	11.84	18.4	5.96	11.75	7.34	2.22
Iran	4.09	4.45	5.9	5.86	1.27	4.72	1.94	0.96
Mexico	7.44	3.45	2.7	4.49	5.34	1.31	2.86	1.02
Italy	3.18	7.10	6.82	6.29	4.50	5.37	2.84	4.24
South Africa	5.60	5.30	7	4.53	3.76	3.66	4.88	3.35
Saudi Arabia	6.86	2.91	11.64	5.18	2.98	3.14	4.33	2.63
Australia	3.55	3.06	2.85	1.98	2.03	1.75	1.71	1.70
Indonesia	3.78	3.09	2.69	6.88	3.22	3.19	4.17	3.04
Brazil	12.05	28.96	21.56	14.12	14.48	8.38	5.30	7.79
France	3.37	3.84	4.44	12.93	3.34	4.35	3.80	3.02
Poland	4.01	3.97	4.38	4.1	3.85	4.19	3.68	3.50
Spain	8.34	14.33	13.01	13.48	9.40	5.63	4.94	4.02
